# Quantitative assessment of cardiac load-responsiveness during extracorporeal life support: case and rationale

**DOI:** 10.1186/1749-8090-5-30

**Published:** 2010-04-27

**Authors:** Antoine P Simons, Marcus D Lancé, Koen D Reesink, Frederik H van der Veen, Patrick W Weerwind, Jos G Maessen

**Affiliations:** 1 Dept. of Cardiothoracic Surgery, Cardiovascular Research Institute Maastricht (CARIM), Maastricht University Medical Center (MUMC), Maastricht, the Netherlands; 2 Dept. of Anesthesiology and Pain Treatment/Dept. of Intensive Care Medicine, Cardiovascular Research Institute Maastricht (CARIM), Maastricht University Medical Center (MUMC), Maastricht, the Netherlands; 3 Dept. of Biomedical Engineering/Biophysics, Cardiovascular Research Institute Maastricht (CARIM), Maastricht University Medical Center (MUMC), Maastricht, the Netherlands

## Abstract

We describe a case of a patient assisted by extracorporeal life support, in which we obtained the dynamic filling index, a measure for venous volume during extracorporeal life support, and used this index to assess cardiac load-responsiveness during acute reloading. While reloading, the obtained findings on cardiac pump function by the dynamic filling index were supported by trans-esophageal echocardiography and standard pressure measurement. This suggests that the dynamic filling index can be used to assess cardiac load-responsiveness during extracorporeal life support.

## Background

The successful use of extracorporeal life support (ELS) for cardiopulmonary assist and as bridge to myocardial recovery has been shown in many cases [1-3]. Unfortunately, weaning from extracorporeal life support often relies on preconceived protocols, if any, and is largely based on trial-and-error and limited data on actual cardiac function.

**Figure 1:**
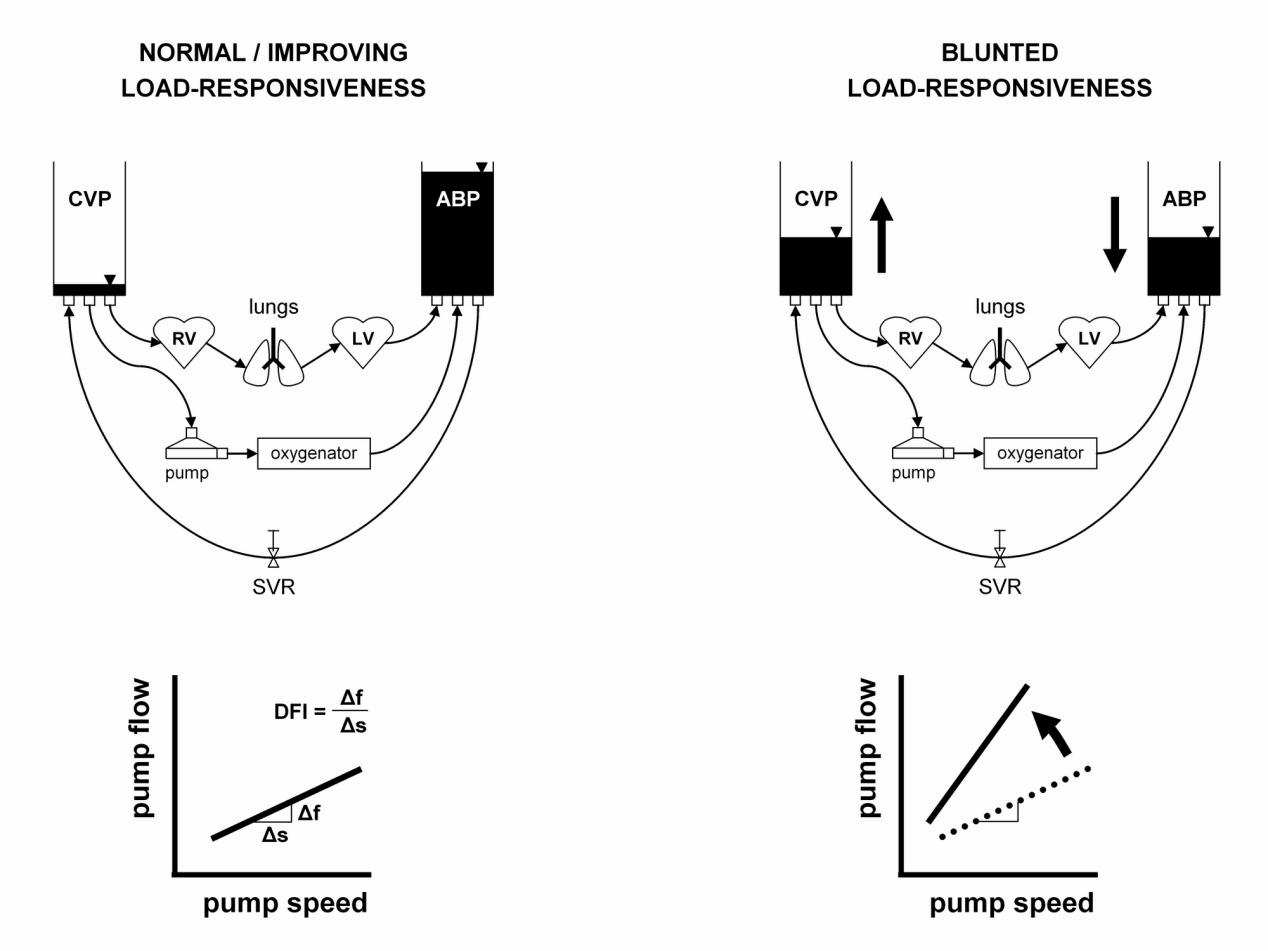
**Rationale behind the use of venous volume measurement (dynamic filling index, DFI) in testing cardiac load-responsiveness**. With acute reduction of pump flow (e.g. from 4 to 2 l/min), the patient remains hemodynamically stable if cardiac load-responsiveness is sufficient (upper left). If cardiac load-responsiveness is blunted, the heart is not able to take over output, and venous volume will increase (upper right). Venous volume modulates the relation between pump speed (s) and flow (f) in the assisted circulation, which can be quantified by the DFI, i.e. the slope of the curve Δf/Δs. The DFI will not increase with diminished ELS flow when cardiac load-responsiveness is good (lower left), but will increase if cardiac load-responsiveness is decreased (lower right). CVP, central venous pressure; ABP, arterial blood pressure; RV, right ventricle; LV, left ventricle; SVR, systemic vascular resistance.

The common procedure to assess cardiac recovery during ELS, is the use of stress tests. These tests induce an increased volume load on the myocardium and address the Frank-Starling response. This response can be visualized using trans-esophageal echocardiography (TEE), and further quantified by measurement of blood pressure and cardiac output [4,5]. Volume loading is achieved by reducing ELS flow, upon which, depending on the degree of recovery, the heart will increase its output and maintain arterial and venous blood pressure (Figure 1). In patients whose heart has not (yet) recovered the response is blunted, which causes low output, decreased arterial blood pressure, and increased venous volume.

Recently, we developed a technique to quantitatively assess venous volume that can be (potentially) drained by the centrifugal pump-based ELS circuit, and introduced the dynamic filling index (DFI, in ml/rotation) for the purpose of optimizing ELS flow [6-8]. DFI measurement uses periodical, transient reductions (-100 rpm) of pump speed, each lasting approximately 10 seconds, superimposed on the steady state pump speed. The resultant changes in bypass flow are used to calculate the DFI as Δflow/Δspeed (Figure 3 in [7]). When venous volume increases the DFI increases, and vice versa. We found that the DFI is more sensitive to detect changes in venous volume than other routinely recorded hemodynamic and pump related parameters, like central venous pressure.

This case shows a patient supported by ELS in which several events of cardiac reloading were performed. In two events, we added DFI measurement to the monitoring of the patient. Before, during, and after reloading, changes in venous volume were quantified using DFI measurement. Under these circumstances, the DFI adequately represented cardiac load-responsiveness as confirmed by TEE.

## Case Presentation

We present the case of a patient with a severe myocardial infarction of the anterior wall. Despite urgent percutaneous coronary intervention and the institution of intra-aortic balloon support, the patient developed cardiogenic shock refractory to medical therapy. To stabilize the patient hemodynamically and provide adequate respiratory support, extracorporeal life support (ELS) was started with an assist flow of 4.2 l/min (Permanent Life Support, Maquet Cardiopulmonary AG, Hirrlingen, Germany).

### Observations during cardiac reloading

**Figure 2:**
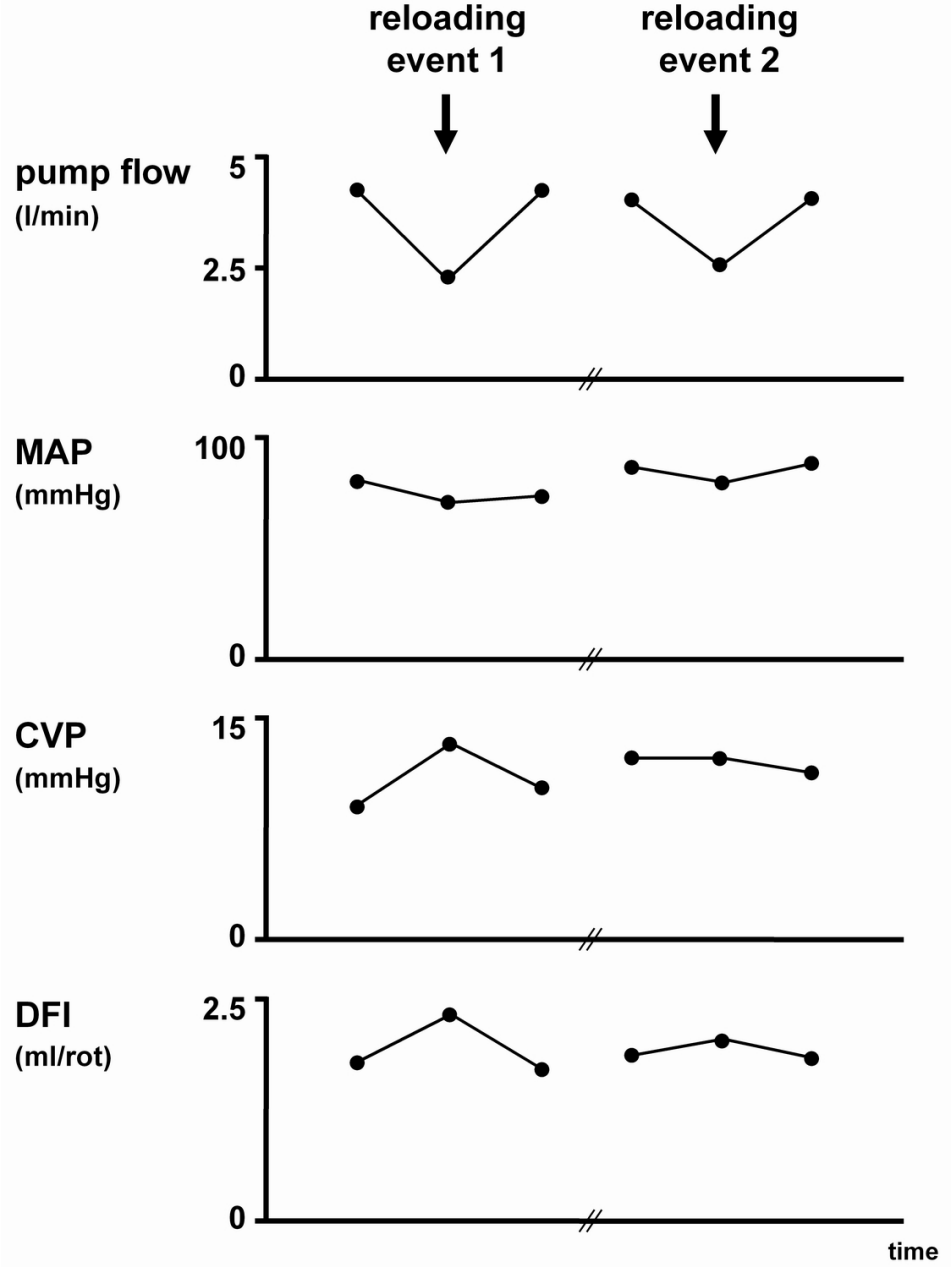
**Hemodynamic parameters during cardiac reloading of the patient supported by extracorporeal life support**. During the first event (left), DFI increased notably with diminished ELS flow. With the second event (right), DFI increased only marginally, suggesting increased cardiac load-responsiveness. MAP, mean arterial pressure; CVP, central venous pressure DFI, dynamic filling index.

We show a less successful and a more successful outcome of on-pump cardiac load-responsiveness. Cardiac reloading was performed by reducing bypass flow by approximately 40-45% with concomitant inotropic support. DFI measurements were performed during full unloading (bypass flow >4 l/min), during reloading (bypass flow ≈2 l/min), and again during subsequent full unloading (bypass flow >4 l/min). The number of DFI measurements for each level of support ranged from 5 to 15, and was 12 on average. The hemodynamic condition and corresponding mean DFI in the two events of reloading are presented in Figure 2.

As support flow was reduced during the first event on day 3, from 4.2 to 2.2 l/min (Figure 2, left), the patient became hemodynamically unstable despite intravenous dobutamine administration. TEE showed poor ventricular function. Radial artery pressure dropped (13%) and central venous pressure rose (44%). The DFI increased by 30%, from 1.8 ± 0.2 to 2.3 ± 0.2 ml/rotation (p<0.001). To stabilize the patient again, flow was increased back to 4.1 l/min. The DFI subsequently decreased by 35%, returning to 1.7 ± 0.2 ml/rotation (p<0.001). The second reloading event described here was started at day four (Figure 2, right). ELS flow was reduced from 4.0 l/min to 2.4 l/min. Parallel TEE indicated improved ventricular wall contractility, and the patient remained hemodynamically stable with a dobutamine infusion set to 5 μg/kg/min. This time, the DFI did not change significantly (p>0.05). Although the DFI readings were not directly included in the evaluation of load-responsiveness, they did independently and quantitatively confirm the increased cardiac load-responsiveness as judged from the TEE and pressure data. After the positive result of this stress test, pump flow was set back to 4.0 l/min to give the heart additional time to recover.

During the next four days, central venous and radial artery pressure indicated improved hemodynamic stability. An improvement of left ventricular function was confirmed by TEE. Based on the observation that cardiac load-responsiveness improved, further DFI measurements were stopped. After three more days of ELS, the patient was weaned from the extracorporeal system with the balloon pump (1:1) in place for additional afterload reduction, and on inotropic support (milrinone 25 μg/kg/h).

## Discussion

This report presents a patient assisted by extracorporeal life support (ELS) in which we demonstrated the use of the dynamic filling index for the quantitative assessment of cardiac load-responsiveness.

The common procedure to assess weanability from cardiac support consists of reduction of device flow, while intensivists, cardiac surgeons, cardiologists and perfusionists are consulted to confirm the rather subjective findings on cardiac recovery. This clinical routine is addressed by Schmid et al. [9], who state that weaning protocols are more exemption than routine, and that patient selection, diagnosis of adequate myocardial recovery, and timing of explant surgery are still unresolved. Pitsis et al. described intermittent unloading and loading of the heart in LVAD-supported patients [10], which can be regarded as cardiac muscle training prior to weaning. In such settings, TEE can be used to visualize cardiac function [4]. TEE, however, provides only a snap shot of the current situation, whereas pressure and cardiac output are measured continuously. In that context, Hoshi et al. described quantitative assessment of cardiac function by continuous measurement of the eccentric displacement of the centrifugal pump impeller [11]. Their measuring principle, however, can only be applied to fully magnetically levitated assist pumps. In all these cases, DFI measurement may support the TEE and hemodynamic observations by providing instant and quantitative information on cardiac pump function. Moreover, DFI measurement is by design applicable to all types of centrifugal pumps. When performed in a regular fashion, the DFI might even enable optimizing the actual weaning process.

In the case presented here, central venous pressure and DFI showed comparable changes in response to ELS reduction and indicated similar changes in cardiac load-responsiveness. Central venous pressure measurement, however, depends on catheter placement, i.e. the location of pressure measurement (jugular vein, caval veins, atrium). When pressure is measured near the inlet of the venous ELS cannula, a change in central venous pressure read-out will, along with hydrostatic pressure, also reflect the rate of suction by the ELS circuit, rather than cardiac preload. Therefore, changes in central venous pressure may in some cases not reflect true changes in cardiac preload. The DFI is a measure of on-pump venous volume [7], and changes in DFI during reduced unloading (i.e. reduced ELS flow) could be a more reliable measure than central venous pressure. We previously found that DFI is more sensitive to small changes in venous volume than pump inlet pressure/central venous pressure [8]. Therefore, if a reliable central venous pressure is unavailable, DFI can be an alternative to assess cardiac load-responsiveness during ELS.

We are aware that we show only a single case in which the DFI was used to support the regular hemodynamic monitoring. More clinical investigations are necessary to establish the additional value of the DFI in the presence of regular ICU parameters. Considering the ease-of-use of DFI measurement and the promising findings in this patient, we intend to study regular DFI measurement in patients assisted by ELS, which, considering the incidence of ELS, will likely require a multicenter study.

## Competing interests

The authors declare that they have no competing interests.

## Authors' contributions

APS, MDL and KDR were responsible for data collection, interpretation of data and writing of the manuscript. FHV, PWW and JGM were involved in critical revision of the manuscript regarding intellectual content and clinical relevance. All authors read and approved the manuscript as written.

## Consent

Informed consent was obtained, and the use of patient data for this case report was approved by the medical ethical committee of the Maastricht University Medical Center.
